# Aggressive Biology of Extra-Nodal NK/T Cell Lymphoma: A Case Report

**DOI:** 10.7759/cureus.48850

**Published:** 2023-11-15

**Authors:** Chanh Huynh, Aman S Sandhu, Matthew Georgy, Rahul Kashyap

**Affiliations:** 1 Oncology, Cancer Care Associates, York, USA; 2 Medicine, Drexel University College of Medicine, Philadelphia, USA; 3 Pathology, WellSpan Health, York, USA; 4 Critical Care Medicine, Mayo Clinic, Rochester, USA; 5 Research, WellSpan Health, York, USA

**Keywords:** nasal cavity, metastasis, enktl, pancreatic involvement, lymphoma

## Abstract

Extra-nodal NK/T cell lymphoma, nasal type (ENKTL) is a rare and aggressive non-Hodgkin lymphoma primarily seen in Asian and South American populations. Diagnosis involves methods like biopsy and molecular testing, with treatment typically combining systemic and radiation therapy. We present the rare case of a 62-year-old female who was diagnosed with localized ENKTL upon initial presentation of nasal congestion. She was started on radiation therapy and responded favorably at first, with decreased congestion and facial swelling. After five weeks of treatment, she developed significant dysphagia and anorexia, which was initially attributed to being a side effect of chemotherapy. After pausing radiation treatment, her condition worsened until she was hospitalized for obstructive jaundice. CT scans and biopsy confirmed metastasis of the cancer to her pancreas. Her condition rapidly declined until she was ultimately sent to hospice care.

## Introduction

Extra-nodal NK/T cell lymphoma, nasal type (ENKTL) is a rare and aggressive form of non-Hodgkin lymphoma (NHL) that typically presents in the nasal cavity and paranasal sinuses. While the disease is more prevalent in Asian and South American populations, it makes up less than 1% of NHL cases across the United States, Canada, and Europe [1]. Patients typically present with nasal obstruction, epistaxis, and discharge. A biopsy is required for confirmation of diagnosis. Immunohistochemistry and molecular testing are performed on the biopsy sample to confirm ENKTL. Most common treatment options involve a combination of chemotherapy and radiotherapy for limited-stage disease [2]. Although ENKTL can present with localized symptoms, it can rapidly spread anywhere throughout the body. In this case report, we highlight the aggressive biology of ENKTL in a 62-year-old female. The peculiar part in this case is that, despite localized presentation and initial favorable response, the cancer progressed from the nasal cavity to the pancreas within the span of three months.

## Case presentation

**Figure 1:**
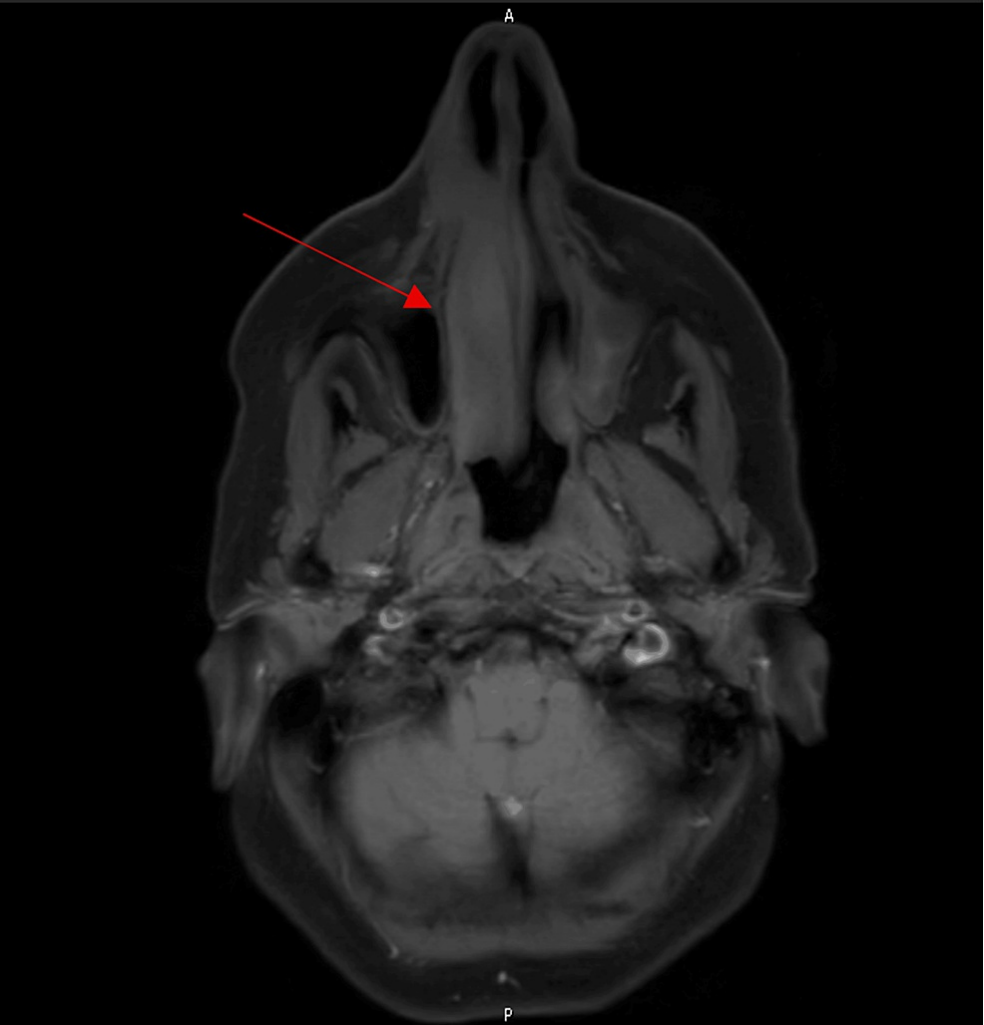
MRI facial showing a soft tissue mass in the nasal cavity

**Figure 2:**
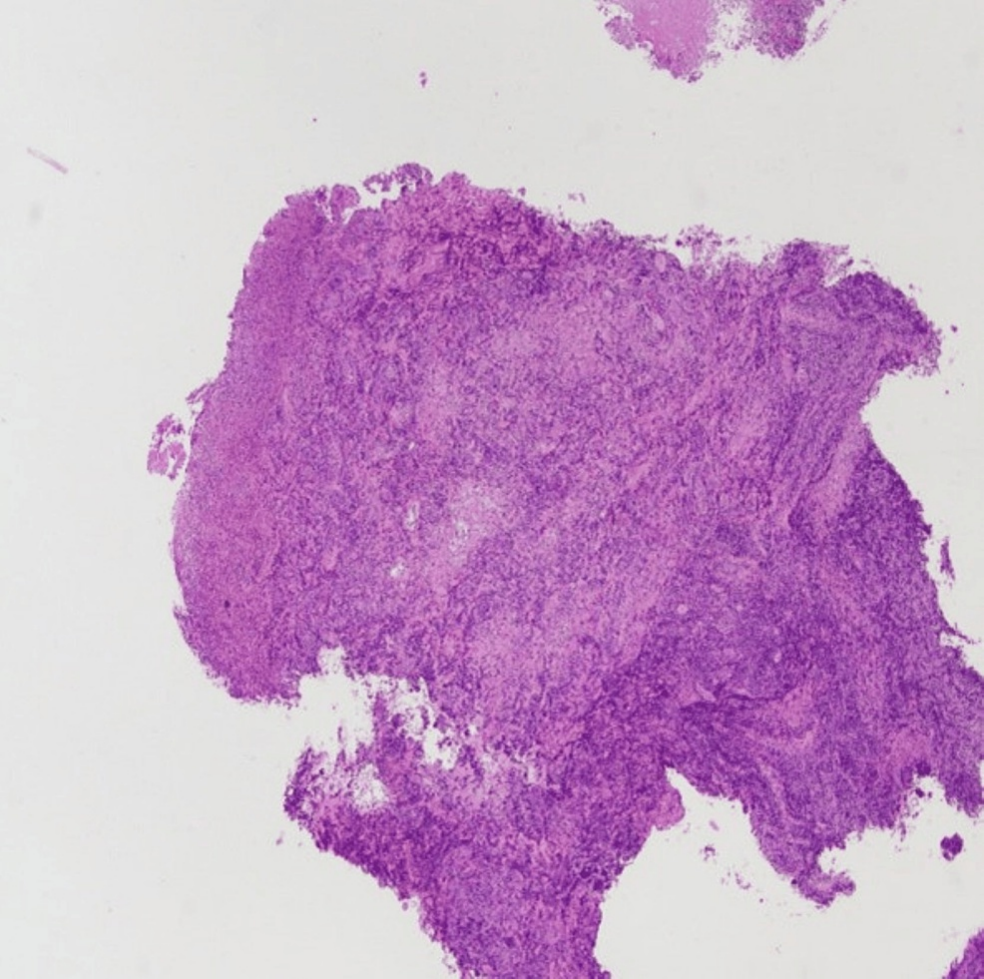
Low-power view of the nasal biopsy showing ulceration and dense infiltrate

**Figure 3:**
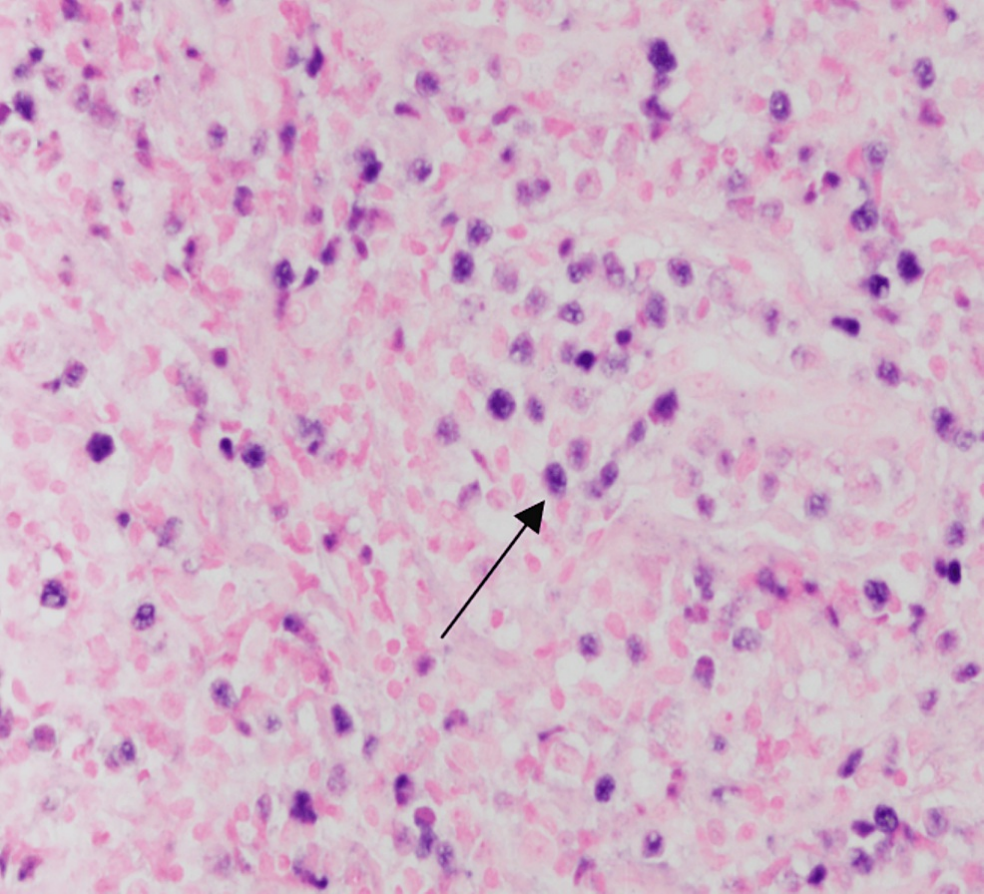
In situ hybridization for EBV (EBER – 400X) showing reactivity within neoplastic lymphocytes EBV: Epstein-Barr virus; EBER: Epstein-Barr encoding region

A 62-year-old woman with no significant medical history presented to her primary care physician in March 2022, with a chief complaint of nasal congestion. She was diagnosed with allergic rhinitis and treated with a course of Augmentin and prednisone. Her follow-up visit with her primary physician in June 2022 was significant for persistent nasal congestion. She was prescribed another course of Augmentin and prednisone. A CT maxillofacial without contrast study was completed, showing indeterminate soft-tissue thickening noted along the anterior nasal cavity, right greater than left. Unfortunately, her symptoms progressed to include facial swelling. A CT MRI facial with and with contrast was completed in August 2022 now showing enhancing soft tissue primarily involving the left nasal cavity extending left of midline. There was involvement of the right greater than the left nasal ridge and preorbital/infraorbital soft tissue (Figure 1). She was referred to Otolaryngology for further evaluation and completed a right nasal cavity biopsy in August 2022 showing extra-nodal NK/T cell lymphoma (Figures 2, 3). Her Epstein-Barr virus (EBV)-DNA polymerase chain reaction (PCR) showed 3400 copies/mL.

The patient was subsequently referred to radiation oncology, and an MRI in August 2022 showed an enhancing soft-tissue mass involving the nasal cavity. Positron emission tomography (PET)/CT showed an intense fludeoxyglucose (FDG)-avid mass extending from the anterior right nostril. There was no significant hypermetabolic disease outside of the nasal cavity.

**Figure 4:**
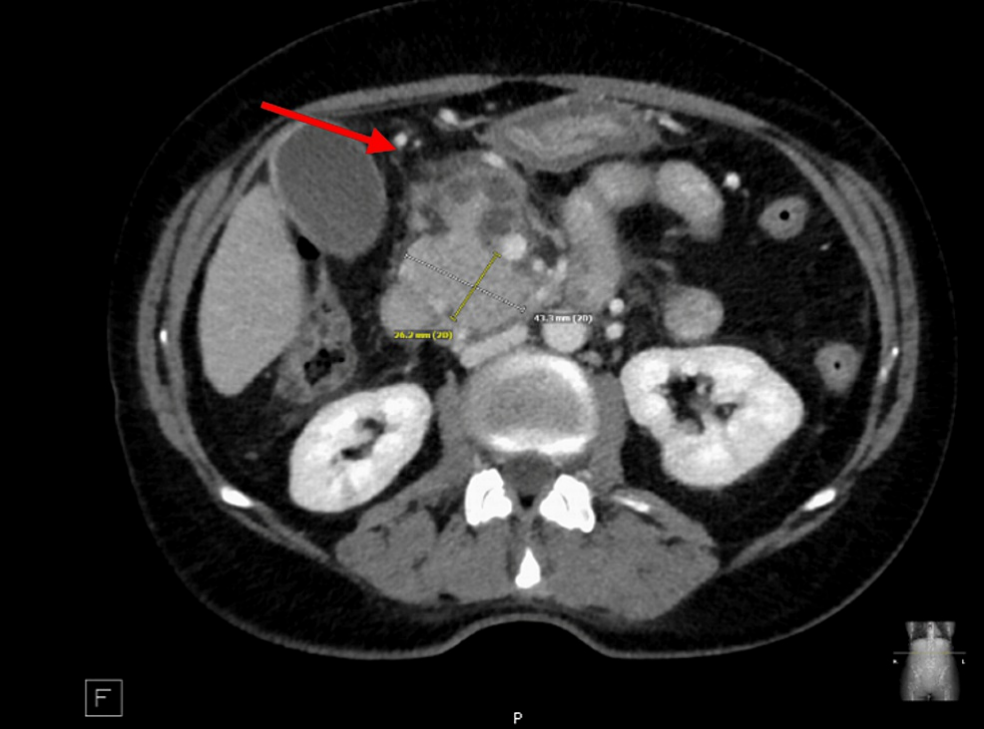
CT abdomen/pelvis showing a 4.3 cm pancreatic head mass

**Figure 5:**
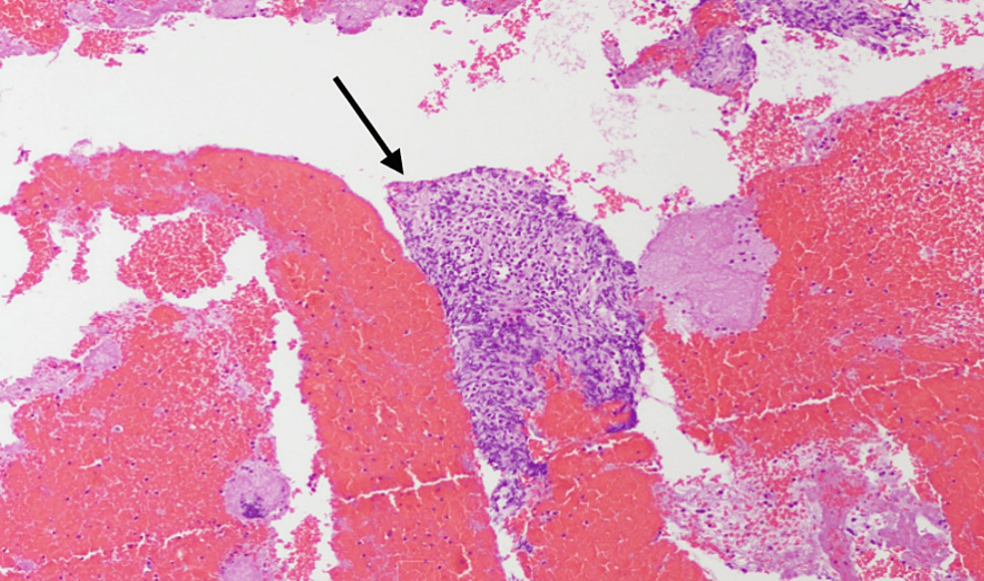
Low-power (100x) pancreatic head FNA showing a tissue fragment with dense lymphoid infiltrate FNA: fine-needle aspiration

A decision was made to start the patient on radiation, due to the rapid primary growth of the lesion, and she was planned for primary radiation to the nasal cavity of 4320 cGy. Initially, the patient had a significant response with improvement in facial swelling and nasal congestion. However, approximately midway through her treatment, 2160 cGy/4320 cGy to the nasal area, she developed significant dysphagia and anorexia. Her radiation treatment was placed on hold, as this was thought to be related to radiation toxicity. However, she was subsequently found to have rising bilirubin and liver enzymes (Table 1) by October 5, 2022. A CT abdomen pelvis with contrast completed on October 17, 2022, showed a soft tissue mass in the region of the pancreatic head measuring 4.3 cm, resulting in moderate intra and extrahepatic biliary ductal dilation (Figure 4). She was hospitalized for obstructing jaundice and subsequently completed an endoscopic retrograde cholangiopancreatography (ERCP) on October 18, 2022, showing a pancreatic head mass. Systemic chemotherapy was not an option at the time due to the rapid disease progression. A CBD stent was placed and a biopsy showed ENKTL lymphoma (Figure 5). The patient’s bilirubin improved after stent placement.

**Table 1:** Patient’s bilirubin and liver enzyme counts across three time periods

	10/5/2022	10/17/22	10/19/22	Reference Range
Bilirubin	0.4 mg/dL	8 mg/dL	1.4 mg/dL	0.1-1.2 mg/dL
AST	55 IU/L	350 IU/L	105 IU/L	8-36 IU/L
ALT IU/L	40 IU/L	315 IU/L	156 IU/L	4-36 IU/L

However, her hospital course was also significant for worsening dyspnea with a CT chest showing multiple bilateral consolidative lung masses. Bronchoscopy showed poorly preserved fragments of fungus. Blood cultures also were positive for *Candida albicans*. Despite aggressive treatment with anti-fungal medications, the patient unfortunately declined rapidly and the decision was made to transition to hospice care.

## Discussion

This case report highlights the aggressive behavior of extra-nodal NK/T cell lymphoma, nasal type. Despite initial localized disease and definitive radiation therapy, the patient rapidly progressed and developed pancreatic metastasis. The case denotes the importance of early diagnosis, prompt treatment, and continuous monitoring of patients with this type of lymphoma [3]. This case also highlights the potential for treatment-related complications. When the patient developed symptoms of dysphagia and anorexia, they were initially attributed as side effects of her radiation treatment.

ENKTL is a rare type of non-Hodgkins lymphoma that primarily infects the nasal and upper respiratory tract. The mechanism of disease is complex and not fully understood. Currently, it is attributed to the abnormal proliferation of natural killer and cytotoxic T cells, leading to damage and inflammation in affected areas. The triggers are unknown, but it has been seen alongside EBV in some cases [4]. Risk factors, such as genetic mutations, environmental triggers, and immune system infection, may be correlated with higher infection rates. Diagnosis and management are complex and typically involve imaging studies, biopsy, and chemo and radiation therapy.

While radiation therapy can be an effective treatment option, this case demonstrates the need for a multidisciplinary approach, including chemotherapy and systemic surveillance. The aggressive pathology of this cancer warrants more extensive monitoring and testing. Regular imaging studies (i.e., CT scans and MRI) are needed to track changes in cancer staging throughout treatment. Although ENKTL has been shown to invade areas beyond the nasopharynx [5], the mechanism of action is still relatively unknown. Pancreatic invasion due to ENKTL is extremely rare, as there are only two other reported cases [6,7]. In Liu et al.’s 2013 study, the infection was due to a primary pancreatic lymphoma (PPL), as the CT scan and otorhinolaryngology examination were negative for nasopharyngeal lymphoma. In Lee et al.’s 2022 study, the pancreatic infection was secondary to testicular infection. Following treatment, the patient complained of epigastric pain, which was originally attributed as a side effect of L-asparaginase treatment. Further imaging and labs confirmed the invasion of tumor cells in the pancreas. These studies denote the importance of continuous monitoring of patients diagnosed with ENKTL.

Further research is needed to better understand the pathology of this disease and to develop more effective treatment strategies to improve patient outcomes.

## Conclusions

In regards to ENKTL, it is important to understand the aggressive nature and potential to invade beyond the presenting region. This case presents the spread of this cancer from the nasopharynx to the pancreas, but virtually any part of the body is susceptible to metastasis. It is important to note that the decline in her improvement was initially attributed to the side effects of chemotherapy. It is important to consider the possibility of metastasis at any point in treatment so that any possible complications can be treated as soon as possible. Extensive monitoring and treatment are required to detect primary and secondary infections.
